# *Drosophila melanogaster* model for transgenerational toxicology: epigenetic and hormonal mechanisms in post-embryonic development

**DOI:** 10.1093/eep/dvag036

**Published:** 2026-10-21

**Authors:** Shadrack Dangabar Apollos, Ummi Ibrahim Hassan, Mardiyya Ibrahim, Ibrahim Malami, Auwalu Garba, Uche Samuel Ndidi, Murtala Bello Abubakar, Aliyu Muhammad

**Affiliations:** National Agricultural Extension Research and Liaison Services (NAERLS), Ahmadu Bello University, Zaria, Kaduna State, 810107, Nigeria; Department of Biochemistry, Ahmadu Bello University, Zaria, Kaduna State, 810107, Nigeria; Department of Biochemistry, Ahmadu Bello University, Zaria, Kaduna State, 810107, Nigeria; Department of Biochemistry, Kaduna State University, Kaduna, Kaduna State, 810107, Nigeria; Department of Biochemistry, Ahmadu Bello University, Zaria, Kaduna State, 810107, Nigeria; Centre for Advanced Medical Research & Training (CAMRET), Usmanu Danfodiyo University, Sokoto, Sokoto State, 840232, Nigeria; Department of Pharmacognosy and Ethnopharmacy, Faculty of Pharmaceutical Sciences, Usmanu Danfodiyo University, Sokoto, Sokoto State, 840232, Nigeria; Department of Biochemistry, Ahmadu Bello University, Zaria, Kaduna State, 810107, Nigeria; Department of Biochemistry, Ahmadu Bello University, Zaria, Kaduna State, 810107, Nigeria; Department of Physiology, College of Medicine and Health Sciences, Sultan Qaboos University, Muscat, 123, Oman; Department of Biochemistry, Ahmadu Bello University, Zaria, Kaduna State, 810107, Nigeria

**Keywords:** *Drosophila melanogaster*, epigenetics, developmental toxicology, transgenerational inheritance, drug development

## Abstract

*Drosophila melanogaster*, a widely used model organism in biology, offers a good platform to study the transgenerational effects of environmental toxicants and pharmaceuticals. Offspring phenotypes are not solely shaped by inherited DNA but also by parental environmental exposures. In this review, we explore how juvenile hormone and ecdysone (key endocrine regulators of insect development) serve as molecular entry points for toxicant-induced epigenetic changes. We detailed how mechanisms such as DNA/RNA methylation, histone modification, non-coding RNAs, chromatin remodeling, and RNA methylation, modulate these hormonal pathways and how they can be inherited across generations. The fly’s short lifecycle, conservation with mammalian systems, and minimal ethical constraints make it ideal for screening developmental and transgenerational toxicities. Leveraging this model can help inform drug development, risk assessment, and public health policy.

## Graphical Abstract

**Graphical Abstract:**
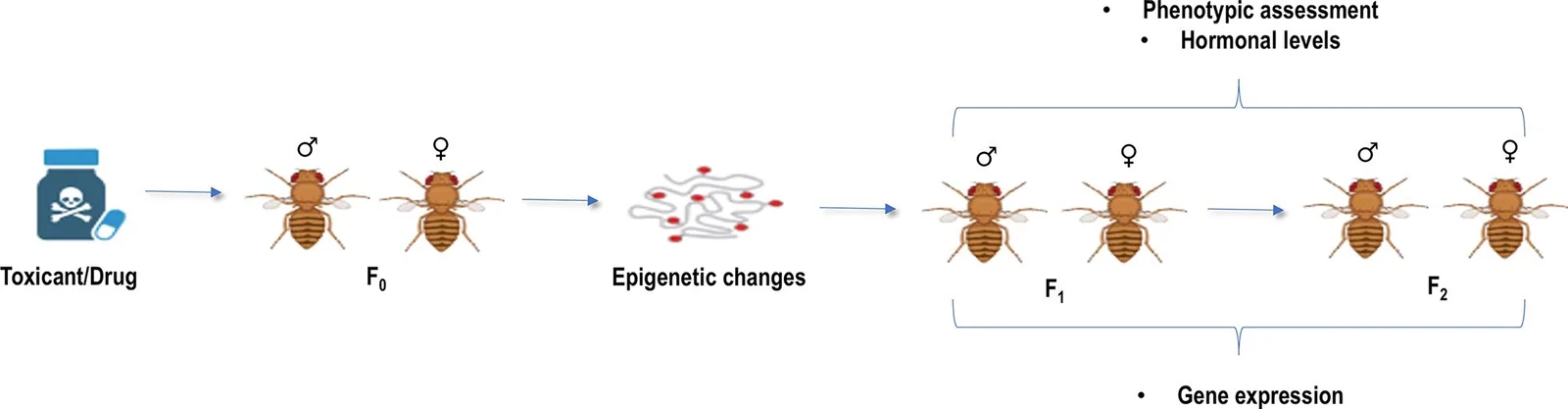

## Introduction

*Drosophila melanogaster* is a dipteran insect which belongs to the family Drosophilidae. It has a rapid life cycle that at 25 °C takes about 10 to 12 days for the adult fly to emerge (Fig. 1), which makes it an alternative model for research in the life sciences. It has met the standards set by the European Centre for the Validation of Alternative Methods (ECVAM), aligning with the principles of the 3Rs, Reduction, Refinement, and Replacement of laboratory animals [1]. The fly was introduced as a model organism in the life sciences some decades ago, where it was largely utilized in the field of genetics [2]. As a model organism, it presents minimal ethical concerns and offers ease of genetic manipulation for studying specific genes under controlled conditions. Since the establishment of Drosophila as a model organism for biological research by Thomas Hunt, the use of the fly has immensely contributed to many findings in the field of biology [3].

**Figure 1:**
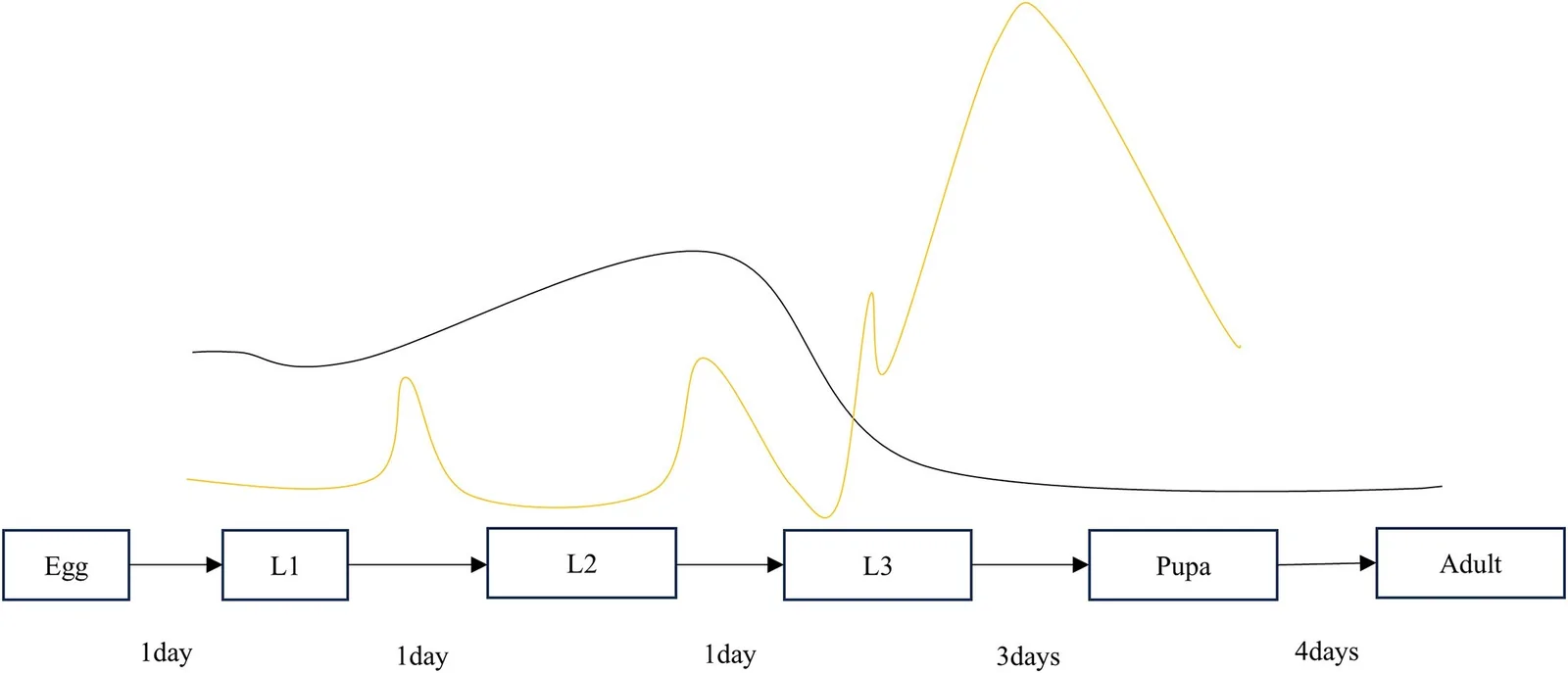
Life cycle of Drosophila melanogaster and the hormonal titers of Juvenile Hormone (JH) and 20 Hydroxyecdysone (20E). High JH levels maintain larval identity and prevent metamorphosis, while spikes of 20E mediate molting. When a critical weight is reached JH levels begin to fall, which occurs during the third instar stage. Heavy growth occurs during the third instar stage until feeding stops and larvae begin to wander to form pupa, a stage triggered by a spike of 20E. A second greater spike of 20E starts the final molt, and due to the absence of JH, the larva molts to the pupal stage. A drop in JH combined with elevated 20E triggers pupation and adult emergence. Where applicable L1 = First insta larva, L2 = Second instar larva, and L3 = Third instar larva.

As an insect that undergoes complete metamorphosis, the fly has four distinct developmental stages (egg, larva, pupa and adult). Based on the key molecular signals that drive the development of the fly, the developmental stages can be categorized into two; embryogenesis and post-embryonic development. Embryogenesis, which is the process of larval formation, takes place within 24 hrs and is driven by massive DNA replication, nuclear formation and the transcription of maternally derived morphogens (bicoid and dorsal) deposited at specific locations in the embryo [4]. The diffusion of these proteins across the embryo forms a gradient resulting in the activation of the transcription of several genes, such as *gap genes, pair-wise genes, segment genes*, and *hox genes* that divide the embryo into segments, regions, and eventually structures [5]. The post-embryonic development encompasses three developmental stages; larva (first, second and third instar larvae), pupa, and adult. The process is largely dependent on the availability of nutrients and hormonal signals, and lasts for approximately 9 to 11 days at 25 °C. After embryogenesis, the first instar larva appears. About 24 hours later, the first instar larva molts into the second instar larva, which also lasts about 24 hours. The second instar larva then molts again into the third and final larval stage, which lasts for about 48 hours. At the start of the third larval stage, the larva reaches a critical size. Toward the end of this stage, it stops eating and starts moving away from the food to become a white prepupa. After that, it enters the pupal stage, which lasts for about 5 to 6 days. Then the adult insect emerges from the pupa [6].

The molting from one larval stage to another during the life cycle of Drosophila is notably controlled by two hormones: juvenile hormone (JH) and ecdysone. JH is known to regulate growth such that it inhibits the transformation from one instar larval state to another, thereby blocking metamorphosis into pupa, while 20-hydroxyecdysone (20E), the active form of ecdysone, helps trigger molting. As shown in Fig. 1, when JH levels are high, 20E mediates changes from one larval form to another. But when JH levels drop, 20E then triggers the changes from larva to pupa, and later from pupa to adult. Hence, the main function of JH is to delay metamorphosis until the right time [7–9].

The significant degree of functional similarity between *D. melanogaster* genes and those found in mammals makes it a viable model for investigating the effect of toxicants in humans. Due to the distinct life stages as a holometabolous insect, Drosophila offers a great opportunity for scientists to study the effects of toxicants and drugs at each of the life stages. It is in this light that “drosophotoxicology” as a term used for the fly (Drosophila) as a model for toxicological study was coined [10]. Furthermore, the short life cycle of the fly makes it suitable for transgenerational inheritance study. Recently, some studies have shown the transgenerational effects of some metals such as cadmium and lead [11–13], a few other compounds such as N-Nitrosodimethylamine and its metabolite formaldehyde, as well as plastic compounds [14–16]. These recent studies illustrate both the range of transgenerational phenotypes detectable in *Drosophila* after ancestral toxicant exposure and the diversity of mechanisms involved. Exposing only the parental (F0) generation to cadmium led wing developmental defects and wing-disc apoptosis; the wing phenotype persisted for at least four unexposed generations, and altered expression of wing-patterning genes (*shg, omb, F-actin, Mekk1*) tracked with heritable shifts in histone H3K4me3, H3K9me3, and H3K27me3 marks, implicating histone methylation as the mediator of the effect [11]. Using a dose-response design, cadmium engaged the Wnt and mTOR signaling pathways as early toxicity responses and impaired fecundity for two to three generations via altered expression of the DNA methyltransferase Dnmt2, with phenotypic (but not molecular) recovery only by the F4 generation [13]. Developmental lead exposure was linked to disrupted neuromuscular junction structure and altered synaptic protein levels (reduced Synapsin, increased Bruchpilot) in F0 flies, alongside genome-wide DNA methylation changes that were transmitted to unexposed descendants, directly connecting a neurodevelopmental phenotype to a heritable epigenetic mark [12]. Beyond metals, the plasticizer DEHP acts as a transgenerational obesogen in a manner dependent on exposure duration, dose, and the sex of the exposed parent: long-term, high-dose paternal exposure increased offspring body weight, while maternal exposure decreased it (indicating that parent of origin shapes the outcome of the same endocrine-disrupting exposure) [14]. In another study, exposing flies to polyethylene and polyvinyl chloride microplastic particles altered offspring sex ratios and fecundity in the F_1_ generation, reduced body size and shortened developmental time in F_2_, despite no measurable change in viral resistance [15]. This suggests that the transgenerational effect is more likely physiological or epigenetic than immune-mediated. On the other hand, comparing the disinfection byproduct N-nitrosodimethylamine (NDMA) with its metabolite formaldehyde, it was found out that although only NDMA was acutely lethal to parental flies, both compounds altered offspring number and induced morphological malformations maintained for at least three generations [16]. This shows that even a non-lethal, non-genotoxic metabolite can produce durable transgenerational developmental toxicity. Collectively, these studies demonstrate that transgenerational phenotypes in *Drosophila* are reproducible across chemically diverse toxicants (metals, plasticizers, microplastics, and disinfection byproducts), are traceable to defined epigenetic marks and physiological pathways, and can persist across multiple generations after exposure has ended. This further supports *Drosophila*’s value as a mechanistically tractable model for transgenerational toxicology.

Organisms are constantly exposed to environmental pollutants, and drugs are constantly manufactured and consumed regularly by both animals and humans. However, there is still a paucity of data on the utilization of the fly for toxicological evaluation during drug development and the possible transgenerational effects of these drugs over time. While the unique lifecycle of the fly makes it highly suitable for transgenerational toxicology, one key challenge is defining the exact molecular interface where toxicants cause lasting transgenerational damage. Hence, in this review, we propose a mechanistic framework where environmental toxicants induce persistent epigenetic alterations e.g. RNA methylation, histone modifications, and non-coding RNA expression that specifically target the biosynthetic and signaling cascades of ecdysone and juvenile hormone, and how scientists can leverage these mechanisms in utilizing Drosophila for the assessment of transgenerational effects of drugs in drug development and monitoring studies. Because these endocrine pathways act as master regulators of post-embryonic development, their epigenetic disruption in the parental germline acts as a molecular bridge in transmitting developmental and physiological toxicities to unexposed subsequent generations.

## Ecdysone and juvenile hormone biosynthesis

Steroid hormones regulate diverse developmental and physiological processes across both vertebrates and invertebrates [17]. In *Drosophila melanogaster*, ecdysone is the primary steroid hormone; it is synthesized from dietary cholesterol or plant sterols to coordinate larval molting and metamorphosis via its active metabolite, 20-hydroxyecdysone (20E) [18]. This biosynthesis occurs in the prothoracic gland (PG) through the sequential action of enzymes encoded by the Halloween genes [19]. The pathway begins with the conversion of cholesterol to 7-dehydrocholesterol (7dC) by Neverland (Nvd) and proceeds through an uncharacterized “Black Box” stage to 3β,14α-dihydroxy-5β-cholest-7-en-6-one (5β-ketodiol). Terminal catalytic steps involve sequential hydroxylations by Phantom (Phm/CYP306A1), Disembodied (Dib/CYP302A1), Shadow (Sad/CYP315A1), and Shade (Shd/CYP314A1) to produce active 20E [17,20].

In contrast, juvenile hormone (JH) is synthesized and secreted by the *Corpus Allatus* (CA) to regulate metamorphosis, reproduction, immunity, aging, and behavior [21–23]. In *Drosophila*, three sesquiterpenoid products; Methyl-farnesoate (MF), JH III and JH bisepoxide (JHBIII) are synthesized via the mevalonate pathway. This pathway involves thirteen enzymatic steps, including early regulation by 3-hydroxymethylglutaryl CoA synthase (HMGS), 3-hydroxymethylglutaryl CoA reductase (HMGR), and farnesyl diphosphate synthase (FPPS), followed by late-step conversions of farnesyl pyrophosphate (FPP) to farnesol, farnesoic acid, and ultimately active JHs via juvenile hormone acid methyltransferase (JHAMT) and epoxidase enzymes [24,25]. Although key components of both pathways are characterized, unresolved gaps remain, such as the exact mechanisms of the ecdysone “Black Box” and the specific regulation of JH intermediates, which future gene editing and omics-driven modeling can help resolve.

## Molecular interactions of ecdysone and juvenile hormone

The progression from larva to pupa and adult in Drosophila depends on a dynamic hormonal interplay between juvenile hormone (JH) and 20-hydroxyecdysone (20E), regulated through complex receptor-mediated transcriptional cascades and epigenetic modulation [8,23 ,26]. Overall, 20E triggers the molting process, while JH determines the nature of the molt. Previous reports have shown that JH maintains juvenile characteristics by antagonizing the actions of 20E and thereby preventing metamorphosis. 20E acts through the nuclear receptor complex composed of the ecdysone receptor (EcR) and ultraspiracle (USP) [7]. 20-hydroxyecdysone (20E) is lipophilic and diffuses directly into target cells, where it binds the ecdysone receptor (EcR), a nuclear receptor that must heterodimerize with Ultraspiracle (USP, the *Drosophila* RXR homolog) to form a high-affinity ligand-binding complex [7,27]. Unlike classical cytoplasmic steroid receptors, the EcR/USP heterodimer is largely nuclear and DNA-bound even in the unliganded state, where it associates with ecdysone response elements (EcREs) in target-gene promoters and recruits corepressors to keep them silent [27,28]. Ligand binding to EcR induces a conformational change in its C-terminal ligand-binding domain which increases EcR/USP affinity for EcREs and exchanges corepressors for coactivators, converting the complex from a transcriptional repressor to an activator and triggering expression of the early ecdysone-response genes (e.g. *E74, E75, BR-C*) [7,29]. JH acts through both intracellular and membrane receptors for signal transduction. While JH intracellular signaling is well characterized, JH membrane signaling is not well understood [30,31]. Methoprene-tolerant (Met), which belongs to the family of bHLH-PAS transcriptional factors, and its paralog Gce have been identified as JH intracellular receptors [32].

Taiman (Tai), a molecule which belongs to the family of bHLH-PAS transcriptional regulators, acts as a co-activator of Met/Gce. It was originally described in Drosophila and later named as the p160/SRC/NCoA-like molecule protein in other insects [33]. Upon JH binding, Met/Gce forms a heterodimer with Tai; then, Nup358 facilitates the movement of the JH intracellular receptor complex into the nucleus, which is dependent on Hsp83 and importin β [34,35]. In the nucleus, the JH intracellular receptor complex binds to E-box-like motifs in promoter regions of JH primary-response genes and then induces their expression. *Krüppel homolog 1* (*Kr-h1*) is a crucial JH primary-response gene that acts as an anti-metamorphic factor. through Kr-h1 binding sites, Kr-h1 represses the biosynthesis of 20E, and thus prevents 20E-induced metamorphosis [26].

The early response genes of ecdysone are BR-C, E74 and E75 [7,36]. These genes each encode a set of protein isomers that serve as transcriptional regulators by binding to the DNA. These primary proteins activate other genes known as late response genes that either directly or indirectly influence metamorphic processes. While the BR-C gene is the main target of 20E and plays a crucial role in the initiation and progress of metamorphosis [37,38], E75A, an isoform protein of the E75 gene serve two functions; one is to repress the activation of BR-C during the early larval stage where 20E and JH are both present [39]. This supports the transition of development from one larval stage to another. Two is to allow the activation of the BR-C gene at the late larval stage when the 20E titer is high and JH is low. The activation of BR-C at this stage triggers the larval-pupa formation [39]. Presently, the cross-talk between 20E and JH is not fully understood as there are still gaps in the knowledge of the JH signaling mechanisms. Fig 2 shows a descriptive overview of the cross-talk between these two hormones, while Table 1 shows the summary of the hormones signaling. However, more studies using advanced techniques will facilitate the complete understanding of this complex interplay. With the current known signaling mechanisms between the two hormones, we proposed Juvenile hormone and ecdysone signaling as a starting point for transgenerational epigenetic studies for three main reasons. First, both pathways converge on a small set of well-characterized transcription factors (EcR/Usp, Met/Gce, Kr-h1, BR-C) whose loci are already known targets of histone modification and miRNA regulation [40–42], giving a direct, testable link between a specific epigenetic mark and a specific transcriptional output. Second, JH and ecdysone titers follow a fixed, predictable schedule tied to defined developmental transitions (Fig. 1), offering natural checkpoints at which to sample epigenetic state and relate it to a precisely timed phenotype Third, because environmental toxicants such as cadmium are already known to disrupt JH and ecdysone titers directly [43], and because ecdysone signaling is mechanistically analogous to vertebrate nuclear hormone receptor signaling[44], epigenetic changes at these loci are more likely to translate to mammalian endocrine-disruption toxicology than changes confined to insect-specific pathways.

**Figure 2:**
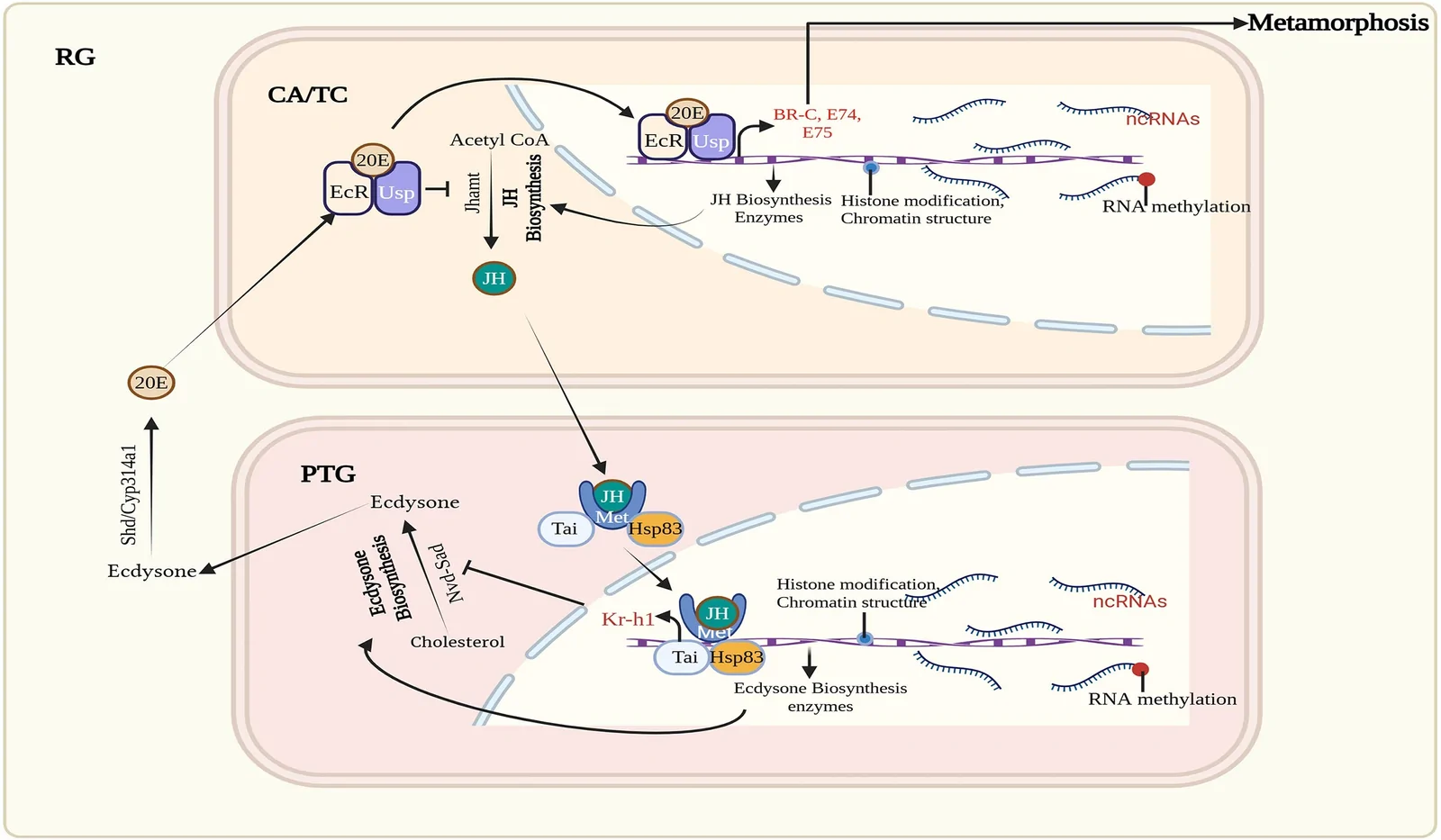
Molecular cross-talk between ecdysone (20E) and juvenile hormone (JH) in Drosophila melanogaster. A schematic illustration of the biosynthetic pathways, receptor signaling, and layers of epigenetic regulation (e.g. histone modifications, DNA/RNA methylation, ncRNAs) that influence development and metamorphosis. 20E binds the nuclear EcR/Usp heterodimer, which remains DNA-bound at ecdysone response elements even in the unliganded state; ligand binding triggers a conformational switch that exchanges corepressors for coactivators, converting the complex from a transcriptional repressor to an activator of early response genes (E74, E75, BR-C) (Yao et al., 1993; King-Jones and Thummel, 2005).Where applicable CA = Corpus allatum (CA), RG = Ring gland, PTG = Prothoracic gland and TC = Target cell. Created in https://BioRender.com.

**Table 1:** Summary of ecdysone and juvenile hormones signaling.

Hormone	Role (s)	Biosynthesis enzymes	Gland	Receptor (s)	Primary response gene (s)	Reference (s)
**Ecdysone**	Molting and metamorphosis	Enzymes encoded by Halloween genes- Nvd, Phm, Dib, Sad and Shd	prothoracic gland (PG)	EcR and Usp	BR-C, E74 and E75	[7,19 ,20 ,36]
**Juvenile hormone**	Status quo maintenance/prevention of metamorphosis	Enzymes of the mevalonate pathway- HMGS, HMGR, FPPS, JHAMT	*Corpus Allatus* (CA)	Met/Gce	*Kr-h1*	[8,9 ,24 ,25 ,26 ,32]

## Development and hormonal signaling in drosophila vs mammals

One feature that living organisms possess is the ability to grow and develop. Regardless of what form an organism starts life, changes either structurally and/or biochemically occur. The sum of all these changes that take place over the lifetime of an organism is referred to as the life cycle. In the case of both insects and mammals, the endocrine system is known to be the master regulator of growth and development. Though the hormones that regulate these processes in insects are not the same as those in mammals, the same architecture exists in both systems. For instance, in mammals, the onset of puberty is manifested by the rise of gonadal steroid hormone production as a result of the activation of the hypothalamic-pituitary-gonadal (HPG) axis. The regular secretion of hypothalamic neuropeptide gonadotropin-releasing hormone (GnRH) induces the production of luteinizing hormone (LH) and follicle-stimulating hormone (FSH) in the pituitary gland. The LH and FSH produced reach the gonads through the circulatory system and stimulate the production of sex steroid hormones. The resulting increase in the levels of circulating sex steroids promotes the pubertal process to acquire adult morphology and physiology [44]. The process of juvenile to adult developmental transition in Drosophila as explained earlier, is also triggered by increased levels of the only insect steroid hormone (ecdysone). As well, the production of ecdysone is stimulated by the pulsatile secretion of the neuropeptide PTTH. This shows that in both systems, hormones are secreted by glands and circulate around the body to promote growth and development.

## Drug development and toxicology

Drug development is a resource-intensive pipeline from target identification, lead optimization, preclinical safety testing, and phased clinical trials, with toxicological evaluation required at every stage before a candidate can advance [45–48]. Furthermore, even when a drug has been approved, risk assessment and labeling are important. In this stage, toxicological studies are conducted to ensure that potential toxicities are communicated to healthcare providers and policymakers [49].

Aside from the promising value Drosophila offers in identifying novel therapeutic targets due to its similarity with mammals, as more than hundred genes implicated in endocrine and metabolic disorders in humans are found to be conserved in the fly [50–52], studies using *D. melanogaster* as a model organism have generated promising data in terms of possible toxicological effects of drugs. A study revealed that ampicillin, one of the frequently used antibiotics, caused a reduction in lifespan and fertility, an increase in *p53* expression, and a decrease in methylation-related genes (*dDnmt2* and *dMBD2/3)* across multiple generations in Drosophila [53]. This last finding requires some interpretive caution. Unlike mammals, *Drosophila* has no functional DNMT1/DNMT3 machinery and essentially undetectable genomic 5-methylcytosine [54]; its single DNMT-family enzyme, Dnmt2, instead methylates cytosine 38 of specific tRNAs, stabilizing them against stress-induced cleavage rather than modifying DNA [55,56]. Similarly, *Drosophila* MBD2/3 retains only a degenerate methyl-CpG-binding domain that has lost the capacity to bind methylated DNA, and instead functions as a DNA-methylation-independent transcriptional corepressor through the Mi-2/NuRD histone deacetylase complex. A decrease in *dDnmt2* and *dMBD2/3* expression following AMP exposure may reflects reduced tRNA-methylation-mediated stress protection and reduced NuRD-complex-mediated chromatin repression, rather than any change in genomic DNA methylation. The relationship between ampicillin and these genes is not yet mechanistically established, but another indirect effect maybe at play. As a bactericidal β-lactam, AMP is likely to disrupt the *Drosophila* gut microbiome, and the resulting dysbiosis and physiological stress could suppress expression of stress-responsive chromatin and RNA-modifying enzymes such as *dDnmt2*, which may further compound the apoptotic (*p53*-driven) germline damage already observed and contribute to the multigenerational decline in fertility. To test this hypothesis, the effects of AMP exposure in axenic and conventionally reared flies can be compared to help clarify whether the epigenetic changes reported are a direct drug effect on the host or a secondary consequence of antibiotic-induced dysbiosis. Another study also examines the transgenerational toxicity and safety of Paracetamol (acetaminophen), The findings show that paracetamol exposure can cause long-term, inherited effects on locomotor activity, with these impacts persisting across generations that were not directly exposed to the drug [57]. The study also reveals that these transgenerational effects are genotype-specific, emphasizing the role of genetic background in shaping the long-term behavioral consequences of pharmaceutical exposure. Due to the similarity of Drosophila development as well as its genome to mammals, this fly can be utilized at each stage of drug development to check for the drug’s safety and also potential genetic effects associated with its use. However, such results may not be exactly replicated in mammals, but may serve as predictions of the potential effects and in making informed public health decisions.

## Epigenetics and transgenerational inheritance in toxicology

The phenotypic features exhibited by organisms are influenced by both genetic and environmental factors, and based on the environmental conditions, a single gene can result in different phenotypes. Not only do current environmental conditions determine the phenotypes of organisms, but also past parental environmental experiences [58–60]. This process in which the gene and the environment interact and result to varying phenotypes regardless of the normal Mendelian gene inheritance, is termed ‘epigenetics’. In a standard definition, epigenetics is defined as “the molecular factors and processes around the DNA that regulate genome activity independent of DNA sequence, and are mitotically stable” [61]. These molecular factors that regulate the genomic process include; DNA methylation [62], histone modification [63], chromatin structure [64], non-coding RNAs [65], and RNA methylation [56].

To elucidate how environmental exposures can impact subsequent generations via epigenetic mechanisms, *Drosophila melanogaster* serves as an ideal model system. A typical experimental workflow involves exposing the parental (F_0_) generation to a toxicant or drug, followed by assessment of epigenetic changes and phenotypic outcomes in the F1 and F2 generations (Figure 3). These assessments may include morphology, fertility, hormone quantification, gene expression (e.g. Kr-h1, EcR), and behavioral phenotyping, helping to establish links between germline-transmitted epigenetic alterations and downstream biological effects (Figure 3).

**Figure 3:**
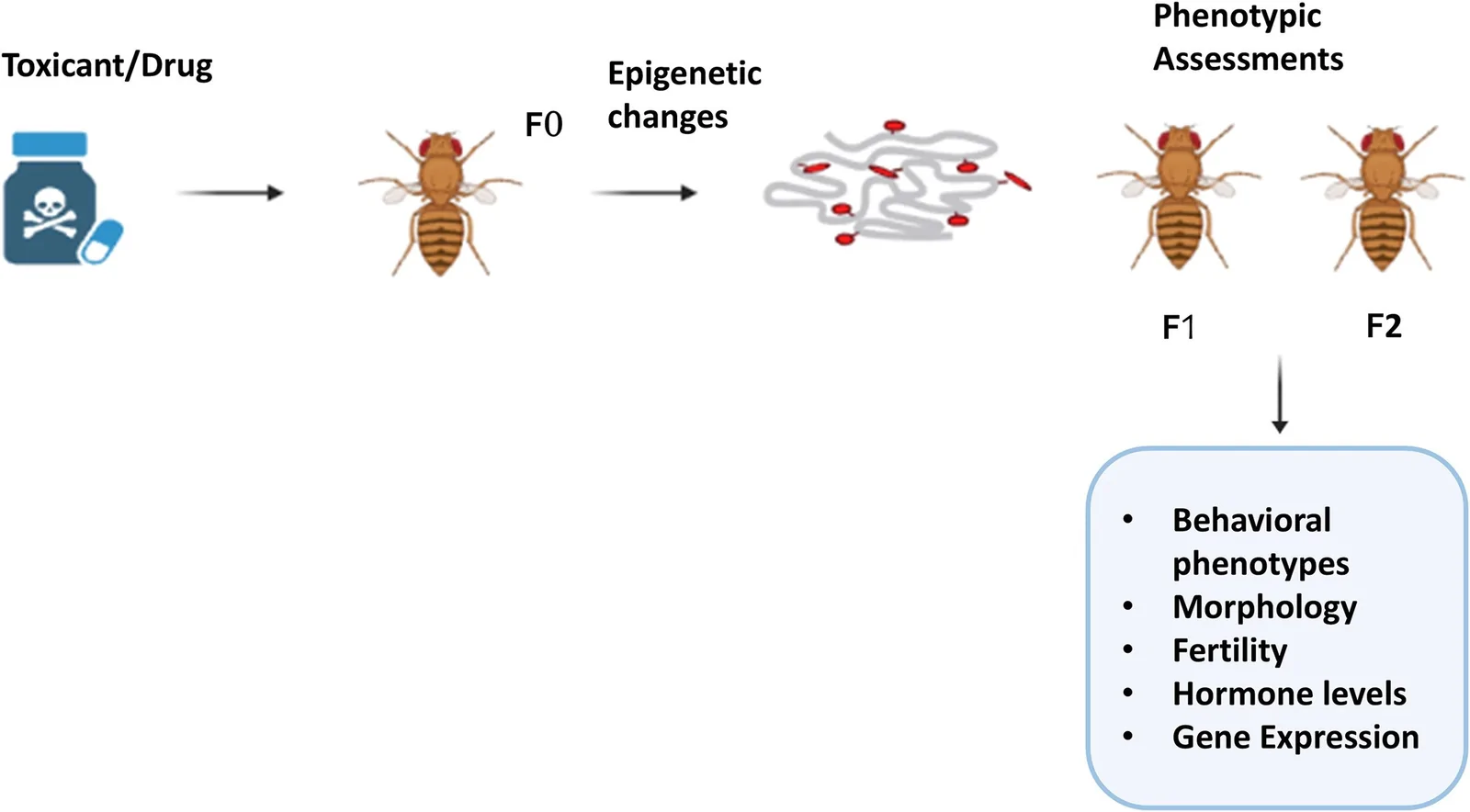
Experimental considerations for using Drosophila in transgenerational studies. Created in https://BioRender.com.

DNA methylation involves adding methyl groups to specific DNA sequences, primarily at CpG sites (cytosine-guanine pairs), resulting in 5-methylcytosine (5mC) [66]. Ten-Eleven Translocation (TET) enzyme family can further modify 5mC into other forms like 5-hydroxymethylcytosine (5hmC) [67]. Generally, 5mC is associated with gene silencing, whereas 5hmC is linked to active gene expression. DNA wraps around histone proteins to form nucleosomes, which can be chemically modified to regulate gene expression. These modifications include acetylation, methylation, ubiquitination, and phosphorylation, among others [68]. These changes can alter chromatin structure, silence genes, or recruit proteins that help or block transcription. Chromatin structure, referring to the 3D organization of DNA and associated proteins within the nucleus, is an epigenetic factor that influences gene accessibility and expression. Chromatin structure can be impacted by other epigenetic mechanisms. For instance, compacted heterochromatin typically represses gene expression and is linked to DNA hypermethylation, whereas less compact euchromatin is associated with active genes and DNA hypomethylation [69]. It is important to note as earlier stated, that unlike in mammals, genomic DNA methylation is exceedingly low or undetectable in most *Drosophila* tissues. Although *Drosophila* possesses a DNMT2 homologue and TET-family enzymes, these appear to act predominantly on tRNA and mRNA cytosines/adenosines rather than genomic DNA [54–56,70]. This fundamental divergence means that DNA methylation-based transgenerational inheritance mechanisms well described in mammals cannot be directly extrapolated to *Drosophila*, and cross-species comparisons of transgenerational epigenetic inheritance should be interpreted with caution. Non-coding RNAs (ncRNAs) are a group of RNA molecules that do not encode proteins but serve to regulate gene expression. Initially thought to only act post-transcriptionally, recent research reveals that various types of ncRNAs, including microRNAs, piRNAs, siRNAs, and long non-coding RNAs (lncRNAs), play a crucial role in controlling gene expression epigenetically [71]. RNA methylation is another epigenetic mechanism that influences gene expression. The most common form is the methylation of adenosine to N6-methyladenosine (m6A), a reversible modification that regulates RNA function after transcription [72,73]. Additionally, cytosine methylation (m3C) occurs in messenger RNA (mRNA) and transfer RNA (tRNA) [74]. These RNA modifications impact RNA structure and gene expression.

The above epigenetic modifications can occur when organisms are directly or indirectly exposed to toxicants, and the traits can be passed from one generation to another. The transmission of these traits over time due to the modification of the germline cells without the direct exposure to toxicants is called epigenetic transgenerational inheritance [61]. Transgenerational inheritance in mammals mean that the effects of the epigenetic modifications as a result of the exposure of the parental generation (F_0_) to a toxicant are transmitted to the third filial (F_3_) generation and beyond, because the primordial germ cells of the second filial (F_2_) generation are present in the first filial (F_1_) generation while in uterus, and are thus exposed to the environment of the F_0_ generation [75]. But in oviparous species, like *Drosophila*, transgenerational inheritance means that the modifications are transmitted to the F_2_ generation and beyond [76]. Hence, the transgenerational effects could be used to indicate the long-term consequences of toxicants.

Couple of studies identified miRNAs (e.g. bantam, miR-8, miR-34 etc.) that epigenetically modify the signaling system of ecdysone and juvenile hormone in Drosophila, where some of their targets include the biosynthesis enzymes such as JHAMT [42], phantom, shade, and disembodied [77], *Kr-h1* [41], as well as their receptors; Met [42] and EcR [78] that serve important roles in the signaling transduction during drosophila development. In terms of histone modification, the histone acetyltransferase complex (dATAC) in *D. melanogaster* has been shown to regulate ecdysone biosynthesis in Drosophila either by acetylating the Ftz-F1 protein, which is a transcription factor that regulates the Halloween genes, or by directly regulating the H4K5 acetylation at the promoter regions of Halloween genes [40]. mRNA methylation as an epigenetic modification found in other eukaryotes, has also been identified in Drosophila [79].

Historically, the viability of *Drosophila* as an epigenetic model was questioned due to its low level of conventional cytosine DNA methylation (5mC) compared to mammals [80]. However, recent toxicological work has proven that the fly relies heavily on highly conserved alternative epigenetic systems; most notably RNA methylation (m^6^A) [81], Polycomb/Trithorax-mediated histone modifications [80], and small non-coding RNA pathways (such as piRNAs in the germline) [82,83] to transmit environmental memories transgenerationally. Therefore, while the specific molecular markers may vary, the fundamental downstream consequence such as disrupted steroid hormone signaling driving transgenerational developmental toxicity, remains highly conserved between flies and mammals [12,84]. In terms of developmental toxicology and transgenerational inheritance using Drosophila as a model organism, a study showed how the exposure of Drosophila to cadmium caused the disruption of juvenile hormone and ecdysone during development, increased the expression of DNA methylation-related genes (dDnmt2, dMBD2/3) in the ovaries (F_0_-F_2_) and testicles (F_0_ and F_1_) and how these effects persisted transgenerationally [43]. Here, Cadmium (Cd) offers a clear mechanistic link between toxicant exposure, hormone regulation, and transgenerational inheritance in *Drosophila*. even though only the first generation was ever exposed to cadmium. The authors linked this to changes in DNA/RNA methylation-related genes in the ovaries and testes, suggesting cadmium leaves a heritable epigenetic “mark” (rather than a DNA mutation) that keeps disrupting hormone signaling in subsequent unexposed generations. A complementary, female-focused study further clarifies the link between Cd exposure and transgenerational hormonal effects. By exposing only, the parental (F_0_) females to Cd, the authors observed ovarian structural damage, increased apoptosis, and reduced fertility, accompanied by suppressed expression of the ecdysteroidogenic genes (*spook, phantom, disembodied, shadow, shade*), the ecdysone receptor (*EcR*), vitellogenin (*vg*), and the juvenile hormone-response gene *Kr-h1*, alongside reduced 20E and vitellogenin titers [85]. Critically, these hormonal and reproductive impairments persisted for two to three generations in F_1_- F_3_ offspring that were never themselves exposed to cadmium. The authors linked this persistence to elevated expression of miR-927 (a microRNA already known to directly target *Kr-h1*) [41] which was itself inherited across the same generations. This finding is significant because it moves the cadmium case study beyond a general association between toxicant exposure and heritable phenotypes, to a specific, testable molecular mechanism: a single, well-characterized microRNA acting on a defined node of the juvenile hormone pathway as the heritable signal linking parental exposure to descendant phenotype. Together, these studies provide a good understanding of how an environmental exposure could be transgenerationally transmitted via the JH/ecdysone axis.

Beyond toxicant-specific studies, foundational work in *Drosophila* has directly demonstrated that epigenetic states can be stably inherited across generations independent of any change in DNA sequence. A study showed that transient heat stress applied to larvae altered chromatin structure at a reporter locus in an ATF-2-dependent manner, and that both the altered chromatin state and the resulting eye-color phenotype persisted for multiple generations in the absence of further stress [86]. Another study similarly established stable *Drosophila* “epilines” that differ heritably in Polycomb-mediated H3K27 trimethylation at a target locus, showing that a transiently induced chromatin state can become self-propagating and transgenerationally stable, with environmental conditions modulating how strongly the associated phenotype is expressed [87]. At the level of small RNAs, maternally transmitted piRNAs can convert an inactive transgene cluster into a stable, paramutagenic piRNA-producing locus capable of silencing homologous sequences in subsequent generations [82], illustrating that small RNA populations, rather than DNA sequence, can themselves serve as the heritable unit. Together, these studies show that *Drosophila* is not merely a convenient model for transgenerational toxicology, but a system in which the molecular basis of epigenetic inheritance (chromatin marks, Polycomb complexes, and small RNAs) can be directly studied, reinforcing the rationale for using the fly to trace how toxicant or drug-induced changes to juvenile hormone and ecdysone signaling might be transmitted to unexposed descendants. There is still a paucity of data regarding the use of Drosophila in assessing the effects of drugs on postembryonic development as it relates to ecdysone and juvenile hormone signaling and the possible epigenetic modification that can be inherited by subsequent generations. Fig 2 highlights the possible epigenetic modifications that can influence ecdysone and juvenile hormone synthesis, while Table 2 shows the epigenetic modifications that have been identified to influence ecdysone and juvenile hormone signaling in *D. melanogaster*. To fully conduct a comprehensive transgenerational inheritance in developmental toxicology using *Drosophila melanogaster*, future work should aim to map tissue-specific epigenomic profiles under toxicant exposure using ChIP-seq, ATAC-seq, and single-cell RNA-seq technologies, to pinpoint the timing and heritability of hormone pathway disruptions. Figure 3 depicts a simple experimental design logic.

**Table 2:** Epigenetic modifications implicated in ecdysone and juvenile hormones signaling mechanisms of *D. melanogaster*.

Epigenetic modification(s)	Target(s)	Reference(s)
miRNAs	JHAMT, *Kr-h1*, Met, phantom, shade, and disembodied, and EcR	[41,72 ,77 ,78]
Histone acetylation	Ftz-F1 and promoter regions of Halloween genes	[40]

## Conclusion

It is evident that the environment and toxicants/compounds to which parental generations are exposed have a great influence on the manifesting phenotypes of the subsequent generations, which are a result of the epigenetic changes to the genome. Juvenile hormones and ecdysone are two important hormones in the postembryonic development of Drosophila. Because these hormones are not proteins, changes in their levels during development (and the epigenetic transgenerational inheritance of those changes) can only be studied in relation to the mechanisms governing their synthesis and signaling.

The future of postembryonic development and toxicology in drug development research using *Drosophila melanogaster* shows great potential, with breakthroughs in understanding the complex relationships between environmental exposures, hormonal signaling, and epigenetic regulations. As advanced technologies such as genome-wide technology (e.g. high-throughput sequencing) that utilize immunoprecipitation, in combination with a two-tiered screening approach to study epigenetic modifications [88–91] continue to emerge, researchers will be able to explain the complex mechanisms that are fundamental to the transgenerational effects of drugs, revealing the long-term consequences on development and health. By incorporating developmental toxicology in relation to the overall hormonal signals and transgenerational inheritance of these changes in drug development using *Drosophila melanogaster* as a model organism, scientists will gain more insights into the interplay between juvenile hormone and ecdysone signaling pathways and their modulation by epigenetic mechanisms, ultimately informing strategies for predicting and preventing developmental toxicity and promoting healthier outcomes for future generations.

In conclusion, due to the advantages such as short generation time, similarity to the mammalian system, little or no ethical concerns, and the available whole genome sequence Drosophila offers, the fly is no doubt a suitable model for transgenerational study in developmental toxicology. Taking advantage of the advanced technologies, scientists can conduct holistic studies with Drosophila and mammalian systems so as to draw insights into the possible extrapolation of such findings in mammals. This will help in making public health decisions and consequently in protecting our future generations.
